# Antenna movements as a function of odorants’ biological value in honeybees (*Apis mellifera* L.)

**DOI:** 10.1038/s41598-022-14354-z

**Published:** 2022-07-08

**Authors:** Hanna Cholé, Alice Merlin, Nicholas Henderson, Estelle Paupy, Prisca Mahé, Gérard Arnold, Jean-Christophe Sandoz

**Affiliations:** 1 Evolution, Genomes, Behavior and Ecology, Université Paris-Saclay, CNRS, IRD, 91198 Gif-sur-Yvette, France; 2 Present Address: Department of Ecology, Evolution, and Behavior, The Alexander Silberman Institute of Life Sciences, The Hebrew University of Jerusalem, 91904 Jerusalem, Israel

**Keywords:** Olfactory system, Social behaviour, Animal behaviour

## Abstract

In honeybees, the antennae are highly mobile sensory organs that express scanning movements in various behavioral contexts and toward many stimuli, especially odorants. The rules underlying these movements are still unclear. Using a motion-capture system, we analyzed bees’ antennal responses to a panel of pheromonal and other biologically relevant odorants. We observed clear differences in bees’ antennal responses, with opposite movements to stimuli related to opposite contexts: slow backward movements were expressed in response to alarm pheromones, while fast forward movements were elicited by food related cues as well as brood and queen related pheromones. These responses are reproducible, as a similar pattern of odor-specific responses was observed in bees from different colonies, on different years. We then tested whether odorants’ attractiveness for bees, measured using an original olfactory orientation setup, may predict antenna movements. This simple measure of odorants’ valence did however not correlate with either antennal position or velocity measures, showing that more complex rules than simple hedonics underlie bees’ antennal responses to odorants. Lastly, we show that newly-emerged bees express only limited antennal responses compared to older bees, suggesting that a significant part of the observed responses are acquired during bees’ behavioral development.

## Introduction

Animal behaviors are the product of their perception of environmental stimuli and of the inner evaluation of their physiological needs. Most behaviors involve movements of the whole animal and reveal overt aversion or attraction toward perceived stimuli^1–4^. However, the study of such behaviors is sometimes difficult in the Lab and cannot easily be conciliated with the constraints of neurophysiological recordings like electrophysiology or brain imaging. Other, more subtle behaviors involve limited movements of small body parts. Yet, they can reveal crucial information about these animals’ physiological and emotional states and their positive or negative evaluation of perceived stimuli. The movements of sensory organs are typically in this category. For instance, eye movements in humans^5,6^ or ear movements in sheep^7–9^ and mice^10^ can provide information about these animals’ attentional and emotional states. While the informational content of the movements of sensory organs appears evident in these animals, it is less established in the case of invertebrates. Yet, a snail’s tentacles quiver toward food odors and this response is modulated by hunger^11^, the snail’s previous experience^12^ as well as the characteristics of the stimulus, like its concentration^13^. Thus, even in invertebrates, analyzing the movements of sensory organs may provide a non-invasive method for acquiring critical information on the perception of stimuli, their biological significance for the animal, as well as how they are influenced by the animal’s physiological state, previous experience, genetic origin, etc.

In honeybees *Apis mellifera*, the antennae are used to sense stimuli from various sensory modalities (olfactory, gustatory, thermosensory, mechanosensory, etc.^14–19^). Bees use their antennae in a great variety of situations. Inside the hive, the bees’ antennae allow them to probe food, wax or other substrates^20–22^ and to communicate with conspecifics, during food exchanges^23–28^ or the waggle dance^29^, thereby conveying a social reinforcement^30^. Outside of the hive, bees use their antennae during foraging, allowing them to detect and learn multisensory cues from flowers (olfactory, tactile, gustatory^31–33^). Therefore, the honey bee antennae are crucial, highly mobile sensory organs, whose movements are essential to their sensory ecology and behavior. However, what type of information about honeybees’ physiological state or their hedonic evaluation of environmental stimuli may be contained in their antenna movements is mostly unknown.

Olfaction is a crucial sensory modality for honey bees, being involved in most stages of their life^34–36^. Bees rely on olfactory cues for instance for taking care of the brood^22,37^ and the queen^38–41^, corpse removal^42–44^, nest defense^45–47^, transferring information about food sources^48^ and learning floral odorants during foraging^49^. All the odorants involved in these tasks bear different biological values for bees, as well as possible differences in attractiveness. Here, we asked whether the movements of honeybees’ antennae may differ among such odorants, and may integrate information about their biological significance and/or hedonic value.

Typically, bees exhibit an antenna scanning behavior in response to sugar stimulation (initiating extension of the proboscis and feeding) or to odorant presentations, characterized by sweeping movements of the antennae towards the stimulus^50,51^. A previous study observed that 4 different odorants triggered scanning antennal responses of different intensity^50^. A forward orientation of the antennae was observed for two main components of the bees’ aggregation pheromones (geraniol and citral) and for a major royal jelly volatile (octanoic—therein called caprylic—acid), whereas an alarm pheromone component (isopentyl acetate) did not induce any change^50^. From the results of all of these studies, one may hypothesize that odorants which have, through innate or acquired mechanisms, a strong positive value for the bees induce strong antennal responses. However, to conclude, a more comprehensive study based on a larger panel of odorants with differing biological values for bees, is missing. To address this question, we used a recently-developed technique based on a motion-capture principle for monitoring antennal movements at a high frequency rate^52^. We analyzed changes in antennal position and velocity in response to a panel of 15 general and pheromonal odorants, with widely differing biological values for bees. In addition, we developed a high-throughput olfactory orientation assay for measuring each odorant’s attractiveness for bees and compared this measure of odorants’ valence to the bees’ antennal responses. Lastly, to understand the ontogeny of these antenna movements, we measured odor-induced antennal responses in newly-emerged bees.

## Results

### Antennal response to odorants

**Figure 1:**
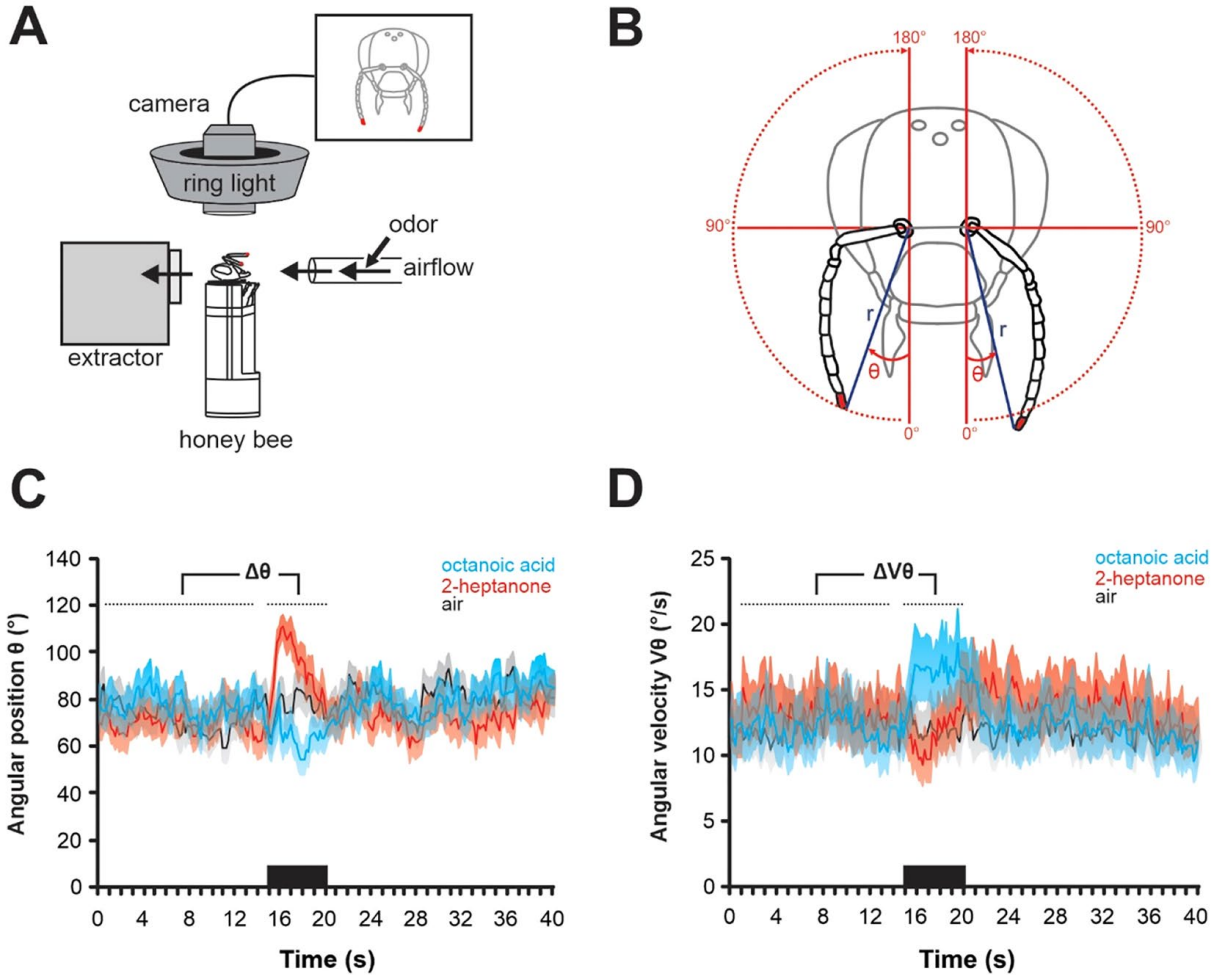
Antennal movements recording. (**A**) Schematic representation of the apparatus for recording bees’ antennal movements. The bee is placed under a camera which detects the colored dots previously applied on bees’ antenna tips. The recording is made in a dark room and the camera is surrounded by a ring light source to control lighting and allow best detection of the color dots. The bee is placed in front of an odor stimulation device. (**B**) Representation of the calculated antennal movement variables: the distance to antenna base (r), the angular position (θ) defined as the angle between the line connecting the antenna tips to their base (r) and an antero-posterior line passing through the corresponding antenna base. The angular velocity (Vθ) is calculated as the angle θ traveled by each antenna during a frame (1/90 s), expressed in degrees per second. (**C,D**) Average recordings of (**C**) antennal angular position (θ) and (**D**) angular velocity (Vθ) in response to the air control (gray line) and to two odorants which induced marked changes: the royal jelly component octanoic acid (blue) and the alarm pheromone component 2-heptanone (red). Curves show average values every 200 ms from the data acquired in the first experiment (N = 24, Fig. 2A,B). Octanoic acid induced a forward motion of the antennae with an acceleration, whereas 2-heptanone induced a backward motion of the antennae with a deceleration. The changes in angular position (Δθ) or angular velocity (ΔVθ) were calculated as the difference between these values during odor presentation (5 s) and before (15 s).

To monitor antennal movements in harnessed honey bees, a camera-based tracking system using a motion capture principle was placed above the bee’s head (Fig. 1A). The upper sides of the bees’ antenna tips were marked with small dots of red acrylic paint, which were tracked by the system at 90 Hz frequency. Bees’ antennae are highly mobile and can move around their socket (henceforth termed ‘antenna base’) from the front of their head to the rear on each side (travelling a ~ 180° angle). Therefore, the position of each antenna tip was best described using polar coordinates, i.e. by a radius (r) and an angle (θ) with the center being the antenna base (Fig. 1B). The radius r was defined as the distance between antenna tip and base while the angle θ was measured from the front (0°) to the back of the bee (180°) via the ipsilateral side (90°). The angular velocity (Vθ) was calculated as the angle θ moved per unit of time (°/s). An olfactory stimulation trial lasted 40 s. After 15 s of an odorless airflow, a 5 s odorant stimulation was applied. Honey bees received stimulations with a panel of 15 odorants chosen for their diversity in terms of emission sources (pheromones emitted by the different members of the hive and/or released in opposite contexts) and possible biological value for bees (floral, fecal, see Table 1). Bees showed a great variety of antennal responses to the presented odorants. Average responses to two remarkable odorants and the air control are shown in Fig. 1C,D (N = 24 bees). While bees’ antennae traveled to the front (Fig. 1C) and increased their velocity (Fig. 1D) when the royal jelly compound octanoic acid was presented, they moved backwards (Fig. 1C) and more slowly (Fig. 1D) in response to the defense compound 2-heptanone. By comparison, the radius (r) did not change much during odorant stimulations (Suppl. Fig. S1).

**Table 1:**
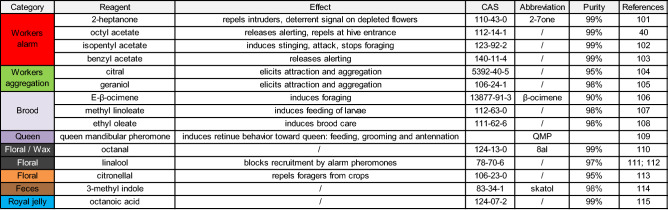
Detailed description of the odorants, their source of production and effect on workers bees. Colors correspond to the legend on the graph: alarm (red), aggregation (green), brood (light purple), and queen (purple) pheromones, and floral odorants (black), repulsive odorant (orange), odor of feces (brown) and of royal jelly (blue). All odorants were obtained from Sigma-Aldrich (Deisenhofen, Germany) except for the queen pheromone QMP, which was a commercial stick purchased as BeeBoost ®, Pherotech, Delta, Canada^40,101–115^.

**Figure 2:**
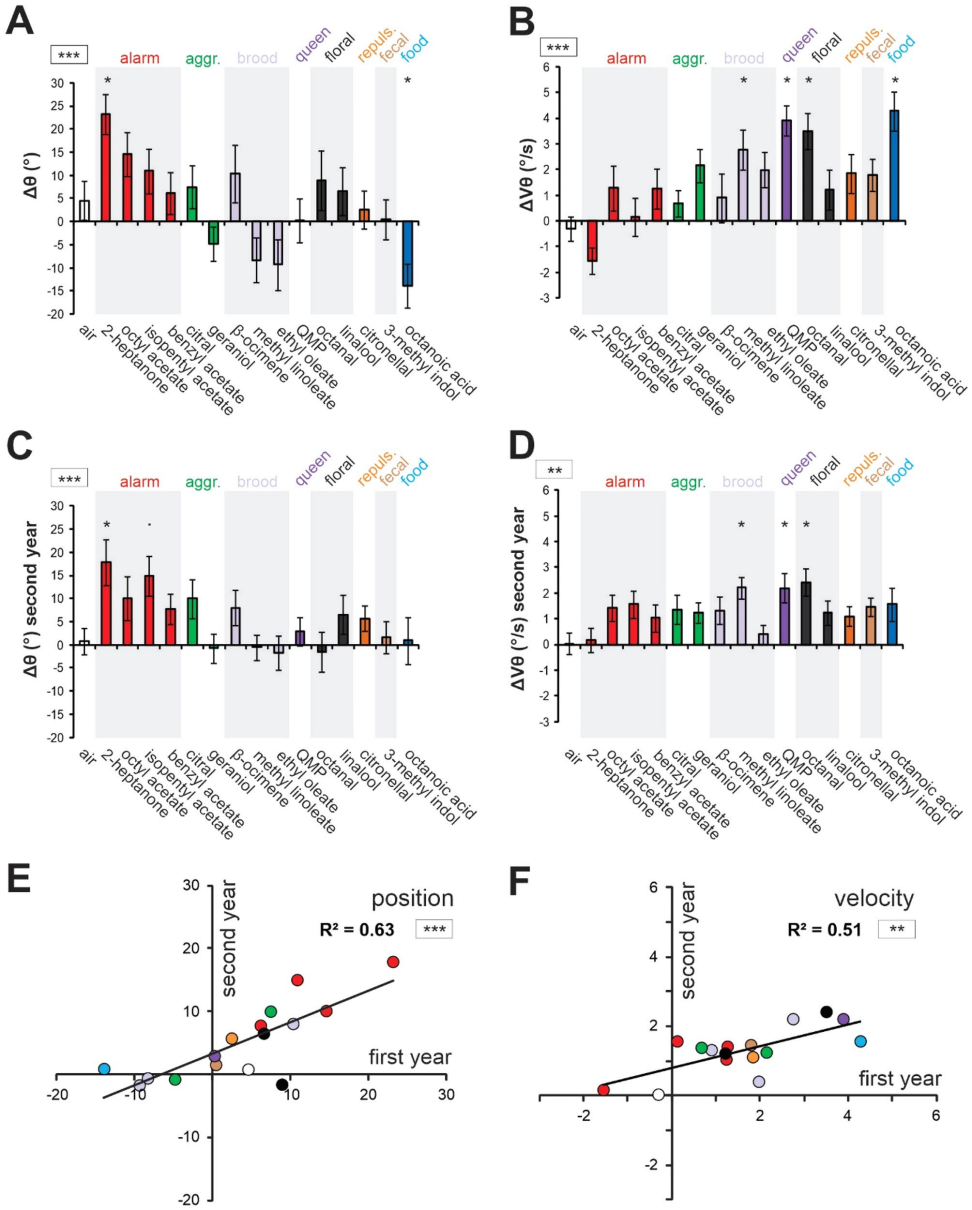
Screening of antennal response to a large panel of odorant. (**A–D**) Histograms showing the change in antennal movements in response to odor presentation (during–before odor) in terms of (**A**,**C**) angular position (Δθ) and (**B**,**D**) angular velocity (ΔVθ). (**A**,**B**) show antennal responses on the first year (N = 24), and (**B**,**C**) show the replication of the experiment on the following year (N = 25). Color code: air control (white), alarm pheromones (red), aggregation pheromones (green), brood pheromones (light purple), queen pheromone (dark purple), floral odors (grey), repulsive odor (orange), fecal odor (brown), and the royal jelly component (blue). Asterisks in the square next to the graph indicate a significant heterogeneity in antennal movements between odorants (RM-ANOVA, ***: p < 0.001). Asterisks on the histograms indicate significant differences in Dunnett post-hoc tests comparing each value to the air control (•: p < 0.1; * = p < 0.05). (**E,F**) Regressions comparing the results of the two experimental years in terms of (**E**) antennal angular position (θ) and (F) angular velocity (ΔVθ). Asterisks in the square next to the graph indicate significance in a Pearson correlation test (***: p < 0.001).

To compare bees’ responses to the different stimuli, we computed Δθ and ΔVθ, defined as the difference in average angular position and velocity between 5 s *during* and 15 s *before* odorant presentation (Fig. 2A,B, N = 24 bees). The fifteen odorants in our panel induced a wide range of antennal responses between the two particular cases illustrated in Fig. 1C,D. Accordingly, we found a significant *stimulus effect* for both angle (Fig. 2A, Δθ: RM-ANOVA, F_15,345_ = 4.45, p < 0.001) and velocity measures (Fig. 2B, ΔVθ, RM-ANOVA, F_15,345_ = 5.32, p < 0.001). Concerning the angle, the antennae went significantly backward (relatively to the air control) in response to 2-heptanone (Dunnett test, p < 0.05) and significantly forward in response to octanoic acid (p < 0.05). Concerning the velocity, bees’ antennae moved significantly faster in response to octanoic acid (p < 0.001), methyl linoleate (p < 0.05), the mandibular queen pheromone (QMP; p < 0.001), and the floral compound octanal (p < 0.001). The only odorant which induced a decrease in velocity was 2-heptanone, but it was not significantly different from the control (air, p = 0.835).

Different odorants thus evoked different antennal responses from adult honey bee workers. We next wondered how reproducible the observed responses are, by testing the same panel of 15 odorants on workers from a different colony, on the next year (Fig. 2C,D). Again, we found a significant *stimulus effect* for the angle (Fig. 2C, Δθ: RM-ANOVA: F_15,360_ = 2.87, p < 0.001), with the same significant backward movement in response to 2-heptanone (p < 0.01), and a tendency for a similar backward movement for another alarm compound, isopentyl acetate (p = 0.0502). Noticeably, on that second year, we observed less numerous forward antennal movements. We also found a significant *stimulus effect* for antennal velocity (Fig. 2D; ΔVθ: RM-ANOVA, F_15,360_ = 2.20, p < 0.01), and, similarly to the first year, a significant velocity increase to octanal (p < 0.01), QMP (p < 0.05), and methyl linoleate (p < 0.05). This time the strong, accelerated forward movement to octanoic acid was not observed.

Despite the few observed differences, antennal responses to odorants were significantly correlated between the two years, both in terms of angle (Fig. 2E, Pearson correlation, R^2^ = 0.63, p < 0.001) and velocity (Fig. 2F, R^2^ = 0.51, p < 0.01). We found no effect of the year, neither for the angle (*year* effect, F_1, 47_ = 0.27, p = 0.60), nor for the velocity (F_1, 47_ = 0.78, p = 0.38). There was however an interaction between *stimulus* and *year* for the velocity (F_15, 705_ = 2.07, p = 0.010), which can be attributed to the fact that velocity differences among odorants were less marked on the second year than on the first (Fig. 2B, D).

**Figure 3:**
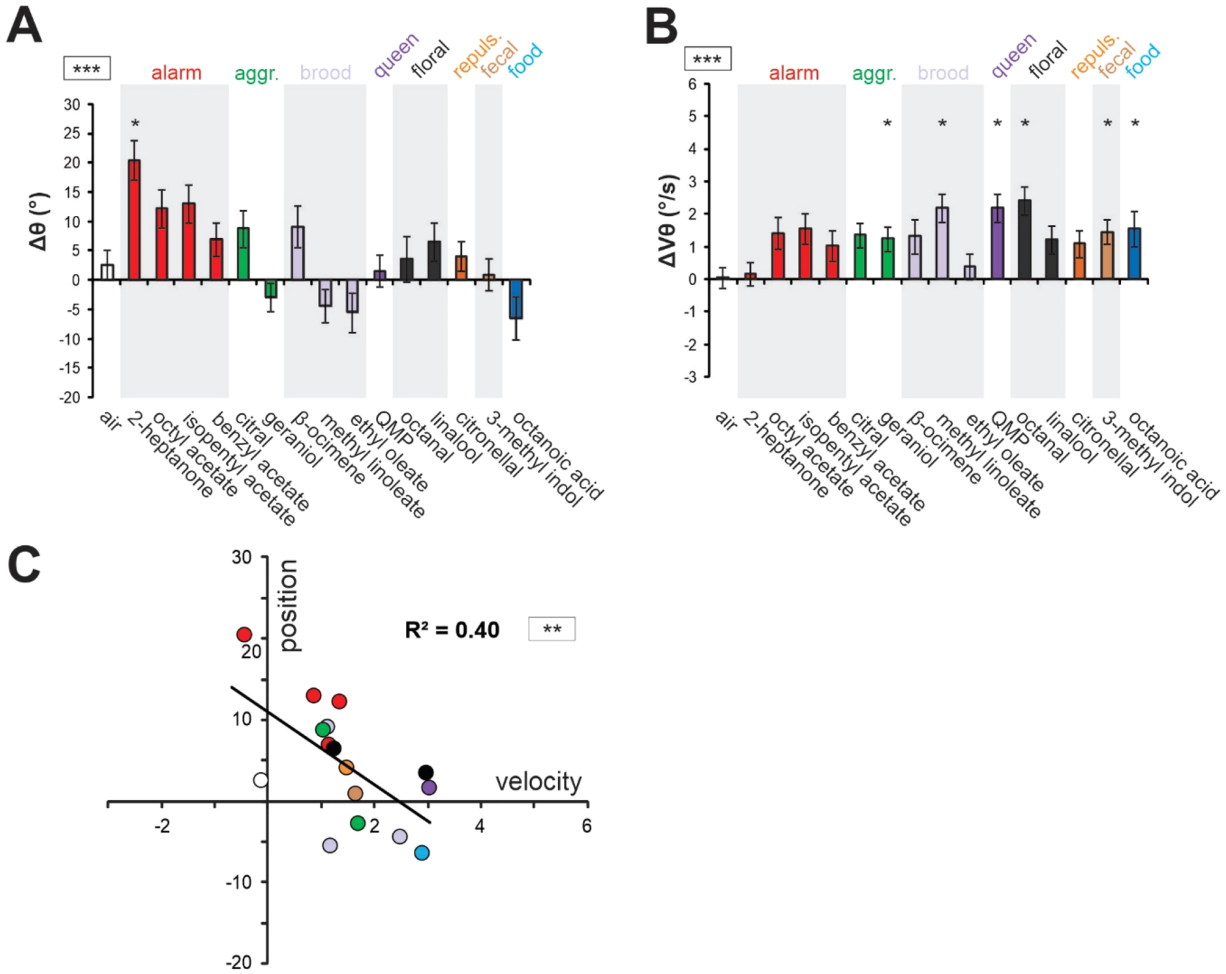
Relationship between changes in antennal angular position (Δθ) and velocity (ΔVθ) in response to odorants. (**A,B**) Histograms showing the change in antennal movements in response to odor presentation (during–before odor) in terms of (**A**) angular position (Δθ) and (**B**) angular velocity (ΔVθ) when pooling data from both experimental years (N = 49). Asterisks in the square next to the graph indicate significant a significant heterogeneity in antennal movements between odorants (RM-ANOVA, ***: p < 0.001). Asterisks on the histograms indicate significant differences in Dunnett post-hoc tests comparing each value to the air control (*: p < 0.05). (**C**) Regressions comparing bees’ responses to the stimuli in terms of antennal angular position (Δθ) and velocity (ΔVθ). Asterisks in the square next to the graph indicate significance in a Pearson correlation test (**: p < 0.01).

Because of the high correlation between the two years’ datasets, they were pooled for further analysis. The resulting united dataset retained strong *stimulus* effects for both angle (Fig. 3A, RM-ANOVA; *stimulus* effect: F_15, 360_ = 2.87, p < 0.001) and velocity (Fig. 3B, RM-ANOVA; *stimulus* effect: F_15, 360_ = 2.20, p < 0.01). We then analyzed the link existing between changes in angular position and velocity in response to the odorants. We found a significant negative correlation between Δθ and ΔVθ (Fig. 3C; Pearson correlation, R^2^ = 0.40, p < 0.01), suggesting that generally, odorants that brought the antennae forward also increased their velocity and conversely, odorants that brought the antennae backward tended to slow them down.

### Attractiveness of the odorants

**Figure 4:**
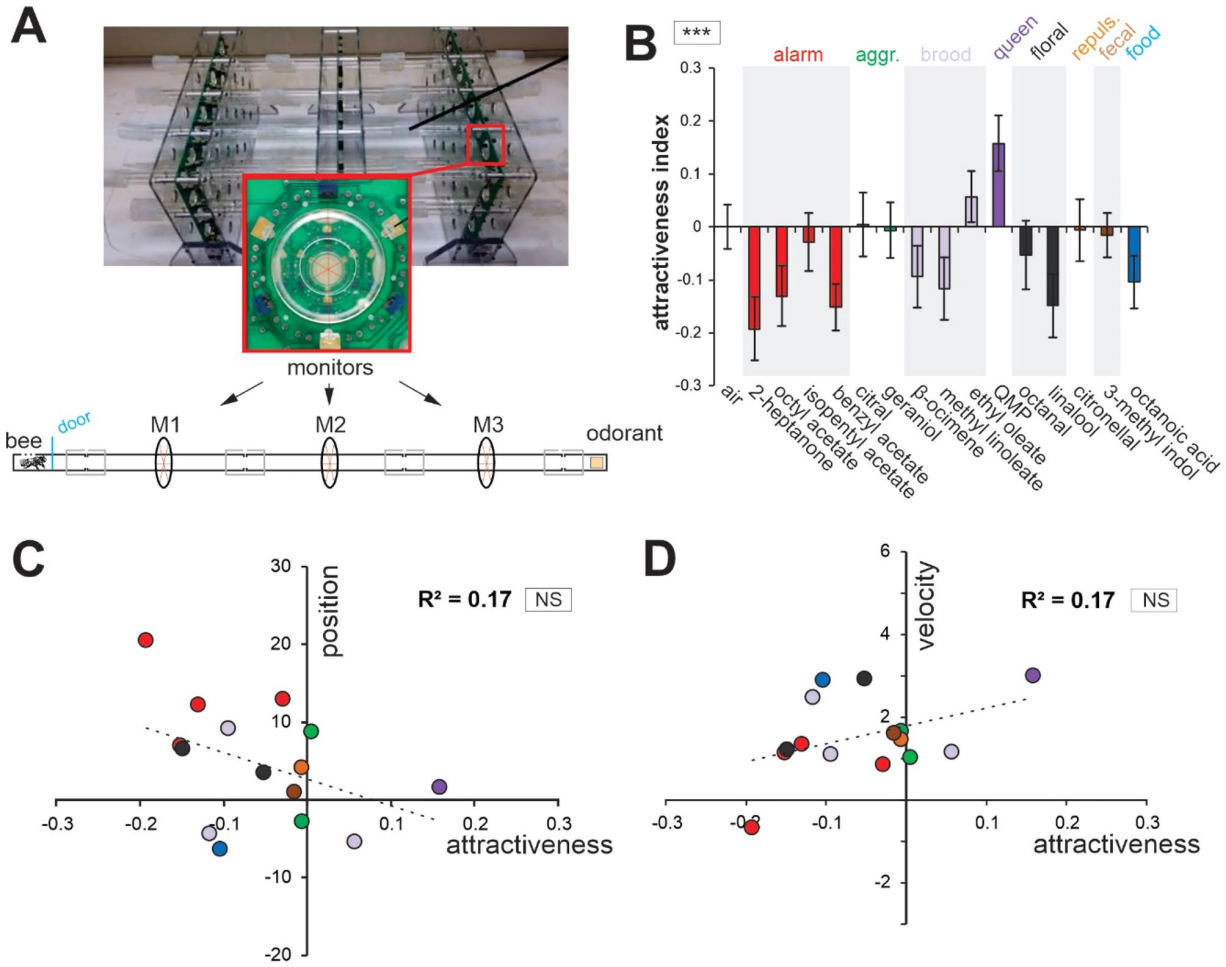
Attractiveness of the odorants and relationship with antennal movements. (**A**) Apparatus for measuring the orientation of 16 bees simultaneously, each confronted to a different odorant. It is made of 16 45 cm glass lines with each 3 equally interspaced infra-red portals (inset). At the start of the experiment, a box containing a worker is placed on one side of each line. A box containing a filter paper soaked with 5 µl odorant solution is placed on the other. The recordings start when opening the doors of the boxes containing the bees and lasts 10 min. From the numbers of passages through the monitors for each odorant and the air control, an attractiveness index is calculated (see text). (**B**) Histograms showing the relative attractiveness index of the odorants. Each bee was used to record the response to only one stimulus: air control N = 46, 2-heptanone N = 40, octyl acetate N = 44, isopentyl acetate N = 42, benzyl acetate N = 43, citral N = 40, geraniol N = 41, β-ocimene N = 43, methyl linoleate N = 40, ethyl oleate N = 42, QMP N = 37, octanal N = 39, linalool N = 40, citronellal N = 42, 3-methyl indole N = 43, octanoic acid N = 39. Asterisks in the square next to the graph indicate significant a significant heterogeneity in the attractiveness index of the different odorants (RM-ANOVA, ***: p < 0.001). (**C,D**) Regressions showing (**C**) the change in antennal angular position (Δθ) or (**D**) angular velocity (ΔVθ) as a function of each odorant’s attractiveness index. NS in the square next to the graph indicates the lack of statistical significance (p = 0.124 and p = 0.126 respectively).

We next asked whether honey bees’ antenna movements relate to their hedonic evaluation of the odorants. To address this question, we developed a high-throughput assay for measuring each odorants’ attractiveness, and measured the movements of about 800 bees in the dark, each confronted to one of our stimuli. The setup consisted in individual 45 cm glass tubes, with a small box containing a bee at one end, and a small box containing a filter paper with a particular odorant (or control) at the other (Fig. 4A). Three equally spaced automatic infrared light portals were positioned along the tube (Trikinetics, Walham, MA, USA, see methods). The movements of the bee through the three portals were monitored for 10 min and an orientation index (OI) was calculated from the number of passages through the monitors:

$$ {\text{OI }} = \, \left( {{\text{M3}} - {\text{M1}}} \right) \, / \, \left( {{\text{M3}} + {\text{ M1}}} \right) $$

M1 being the monitor close to the bee’s initial position and M3 the monitor closest to the odorant box. The control group was presented with an odorless box. To measure the relative attractiveness and repulsion of each odorant, the average orientation index of the blank control group was subtracted from that of each odorant. This way, a positive attractiveness index (AI) indicated an attracting odor whereas a negative index indicated a repellent odor (Fig. 4A). In agreement with the widely differing sources and inferred biological values of our odorants (see Table 1), we observed a significant heterogeneity in the AIs of the different odorants (Fig. 4B: ANOVA; *stimulus* effect: F_14, 600_ = 2.71, p < 0.001). Only two odors produced by the queen and the brood showed a positive attractiveness index: QMP and ethyl oleate. The aggregation pheromone compound geraniol produced only a slight attraction. The rest of the odorants were neutral or acted repulsively.

We then plotted the two variables of antennal movements, Δθ and ΔVθ, as a function of attractiveness values (Fig. 4C,D). Weak trends seemed to emerge, with odorants’ attractiveness suggesting forward and quicker antenna movements. However, possibly due to the limited number of odorants, we found no significant correlation between AI and Δθ (Fig. 4C, Pearson correlation, R^2^ = 0.17, p = 0.124) or between AI and ΔVθ (Fig. 4D, R^2^ = 0.17, p = 0.126). Note that this result was the same when only the first year’s antenna movement dataset (Δθ vs AI: R^2^ = 0.15, p = 0.14; Δθ vs AI: R^2^ = 0.19, p = 0.10) or only the second year’s antenna dataset (Δθ vs AI: R^2^ = 0.16, p = 0.14; Δθ vs AI: R^2^ = 0.07, p = 0.34) were used. We conclude that honeybee’s antennal movements to odorants cannot be directly predicted by a simple measure of odorant attractiveness.

### Concentration influence on the antennal responses

**Figure 5:**
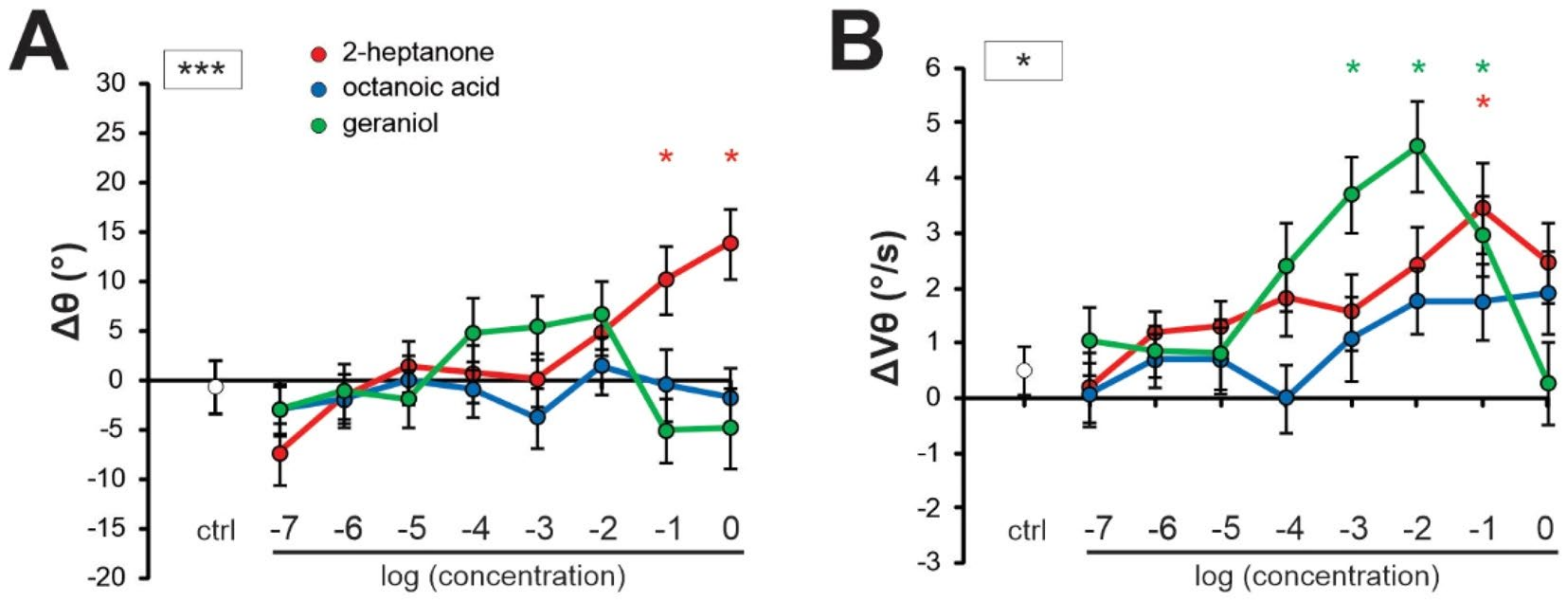
Influence of odorant concentration on antennal movements. (**A,B**) Curves showing the change in (**A**) antennal angular position (Δθ) and (**B**) velocity (ΔVθ) in response to increasing concentrations (from 10^–7^ to 10^0^) of three odorants diluted in mineral oil (N = 43). Ctrl: average response to 4 control stimuli. Asterisks in the square next to the graph indicate significant interactions between *stimulus* and *concentration* (RM-ANOVA, *: p < 0.05; ***: p < 0.001). Asterisks on the graph indicate significant differences in Dunnett post hoc tests comparing each concentration to the control (*: p < 0.05, red: 2-heptanone, green: geraniol).

We next wondered how odorant quantity affected antennal responses and attractiveness measures. Because the same volume of pure odorant was used for almost all stimuli, the absolute concentration in the air flow depended on its vapor pressure, which was different among our odorants (Suppl. Fig. S2A). Indeed, we found that antennal position (Δθ) was significantly correlated to odorants’ vapor pressure, i.e. more volatile odorants (with a higher vapor pressure and accordingly presented at higher concentration) tended to induce backward antenna movements (Suppl. Fig. S2B, Δθ vs VP_log_, Pearson correlation, R^2^ = 0.63, p < 0.01). By contrast, neither antenna velocity (Suppl. Fig. S2C, ΔVθ vs VP_log_, R^2^ = 0.20, NS) nor attractiveness correlated with odorants’ vapor pressure (Suppl. Fig. S2D, AI vs VP_log_, R^2^ = 0.04, NS). Based on these observations, we next tested how odorant concentration may affect antenna movements. We selected three odorants that induced remarkable changes in antennal movements in previous experiments: the defense compound, 2-heptanone, the aggregation compound geraniol, and the royal jelly odorant, octanoic acid (Fig. 5). They were presented to the bees at eight different concentrations ranging from 10^–7^ to 10^0^, in increasing order.

We observed very different evolutions of antennal responses to the three odorants as a function of concentration. Concerning antenna position (Δθ), we found a significant general effect of the *stimulus* (RM-ANOVA, F_2,82_ = 3.45, p < 0.05), of the *concentration* (F_7,287_ = 2.24, p < 0.05) and a highly significant interaction between both variables (F_14, 574_ = 3.25, p < 0.001). Likewise, for antenna velocity, there was a significant effect of the *stimulus* (RM-ANOVA, F_2, 82_ = 5.46, p < 0.01), of the *concentration* (F_7,287_ = 5.59, p < 0.001) and a significant interaction (F_14, 574_ = 1.98, p < 0.05). Analyzing each odorant separately, we found that antennal position changed significantly according to the concentration of 2-heptanone (Fig. 5A; *concentration* effect: F_8, 336_ = 5.26, p < 0.001), with significant differences with the control at the two highest concentrations (Dunnett test: 10^0^ p < 0.01; 10^–1^ p < 0.05). Antennal position also changed significantly with geraniol concentration (F_8, 336_ = 2.15, p < 0.05), but there were no significant differences relatively to the control. Octanoic acid concentration did not affect antennal position (F_8,336_ = 0.31, NS). Antennal velocity changed significantly according to the concentration of 2-heptanone (Fig. 5B; *concentration* effect: F_8,336_ = 2.55, p < 0.05), with a significant difference with the control at the 10^–1^ concentration (Dunnett test: p < 0.01). Antennal velocity also changed significantly with the geraniol concentration (F_8,336_ = 5.86, p < 0.001), with significant differences with the control at 10^–3^ (p < 0.01) and 10^–2^ concentrations (p < 0.001) and a tendency at 10^–1^ (p = 0.051). There was no effect of octanoic acid concentration on antennal velocity (F_8,336_ = 1.49, NS). We conclude that odorants’ concentration affects antenna movements.

### Antennal responses in newly emerged bees

**Figure 6:**
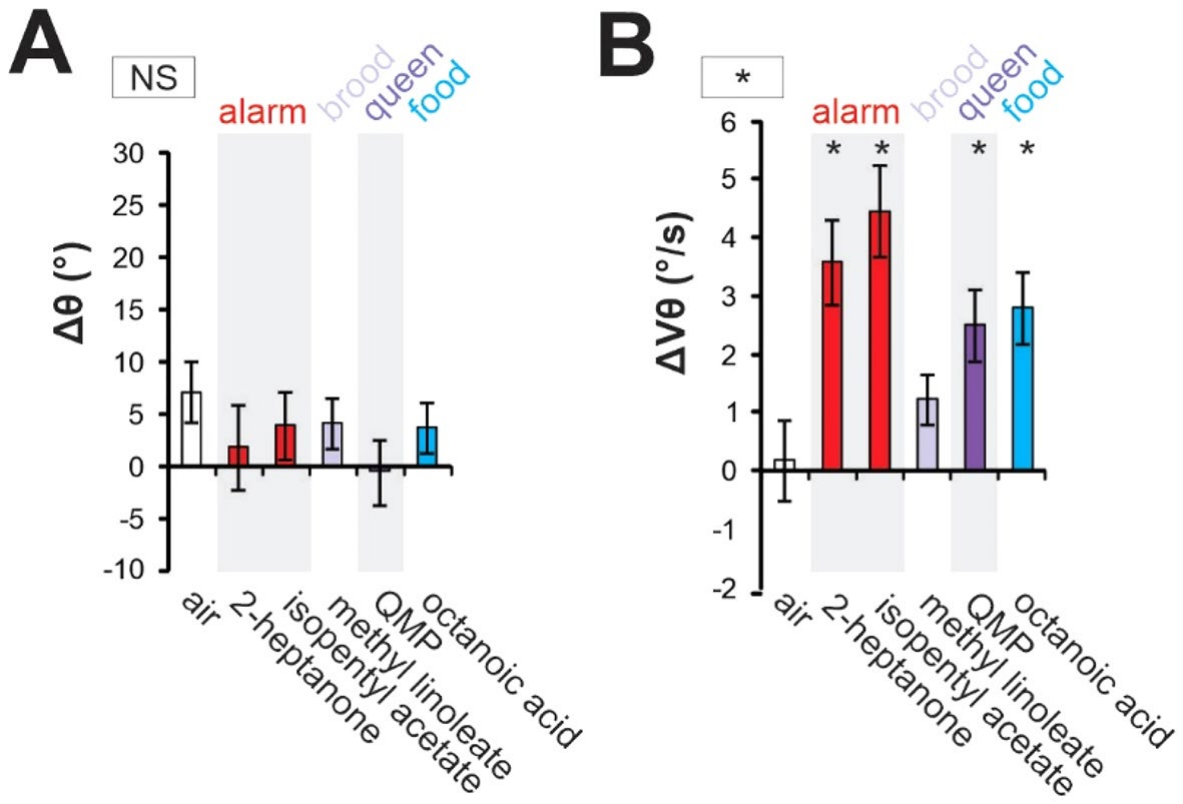
Odor-induced antennal responses in newly emerged bees. (**A,B**) Histograms showing the change in antennal movements in response to odor presentation (during–before odor) in terms of (**A**,**C**) angular position (Δθ) and (**B**,**D**) angular velocity (ΔVθ). Stimuli include an air control (white), two alarm pheromone components (red), one brood pheromone component (light purple), the queen mandibular pheromones (dark purple) and a major component of the royal jelly odor (blue). The asterisk in the square next to the graph indicates a significant heterogeneity among odorants (RM-ANOVA, *p < 0.05). NS: non-significant. Asterisks on the graphs indicate significant differences in Dunnett post-hoc tests comparing each value to the air control (*p < 0.05).

To further evaluate the possibility that antennal responses to odorants are acquired in the course of honeybees’ adult life, we analyzed them in newly emerged bees. Bees that had emerged from their comb cell in the last 24 h were stimulated, together with an air control, with a selection of five olfactory stimuli which had induced contrasted responses in older bees in the previous experiments: two alarm/defense pheromones (2-heptanone, isopentyl acetate), one brood pheromone (methyl linoleate), the queen pheromone (QMP), and the royal jelly odor (octanoic acid). The changes in angular position (Δθ) and velocity (ΔVθ) recorded during odorant stimulations are presented in Fig. 6. Newly emerged bees did not orientate the antennae in response to the different odorants, as no contrast among stimuli appeared for Δθ (*stimulus* effect: F_5, 120_ = 0.81, NS). However, newly emerged bees increased antenna movements when odorants were presented, with a significant heterogeneity observed among stimuli for ΔVθ (*stimulus* effect: F_5, 120_ = 7.54, p < 0.001). Almost all odorants tested induced an increase in velocity (2-heptanone, isopentyl acetate, octanoic acid, QMP; Dunnett tests, p < 0.05; exception methyl linoleate, NS).

## Discussion

In this study, we observed odor-specific antennal movements to a range of pheromones and general odorants with different biological values for honey bees. Bees’ responses recorded from two colonies, on two different years, were correlated, showing that antennal responses to odorants are reproducible. Generally, odorant-induced changes in antennal position and velocity were correlated, so that the more the antennae were brought to the front, the more quickly they moved. Building an original olfactory orientation setup, we observed clear differences in tested odorants’ attractiveness to the bees. However, odorants’ attractiveness, as measured in our setup, did not correlate significantly with either antennal position or velocity measures. Lastly, we show that the antennal responses of newly-emerged bees are limited compared to older bees. While the tested odorants induced an acceleration of antenna movements like in older bees, they did not produce any change in antenna position.

We included in our experiments a few odorants that had been tested in previous recordings of antenna movements^50,51^. In Erber et al. (1993)^50^, bees’ antenna movements were recorded thanks to two photodiodes, each one located in front of one of the bees’ antennae (at an approximately 45° angle in our coordinate system, see Fig. 1B therein). An ‘antennal response’ in this study corresponded to an increased frequency of antennal passages on the diodes during odor presentations. Bees ‘responded’ to geraniol and citral (aggregation pheromone components) and to octanoic acid (therein termed caprylic acid, a major royal jelly volatile) but not to isopentyl acetate (alarm pheromone). In this previous work, it was however not possible to know if bees kept their antennae more to the front and/or increased their antennal scanning velocity during a response. The use of a camera-based system in our study allowed disentangling these effects. Fitting with Erber et al. (1993)^50^, octanoic acid produced in our recordings accelerated movements to the front. Citral and geraniol slightly increased antennal speed (about 1°/s), even if they produced contrasted changes in antenna position (Fig. 3A,B). Lastly, although isopentyl acetate induced an increase in antennal speed (Fig. 3B), it brought the antennae strongly to the back of the bees’ head (Fig. 3A), explaining the lack of response in Erber et al. (1993)^50^. We acknowledge that, like formerly described systems^51,53,54^, our recordings focused on the measure of antennal movements in the transverse plane of the honey bee head, thus in two dimensions. This persisting choice in the studies of antennal movements is due to the observation that most of the bees’ antennal movements take place in this plane. We are therefore confident that the changes in antennal movement observed in the present study represent a prominent and relevant part of the bees’ antennal behavior during odor scanning. Developing a three-dimensional recording strategy by using two or more motion capture systems placed around the bee’s head and by temporally synchronizing their dataflows is possible and some recent efforts were made in this direction^55^.

Thanks to the use of a wide odorant panel, we showed that odorants induce diverse antennal responses, with both forward and backward movements, and both increased and decreased velocities. Interestingly, position and velocity changes in response to odorants were correlated (Fig. 3C) and bees’ antennal responses could roughly be separated in two groups: fast-forward movements and slow-backward movements. It is possible that the observed correlation between position and velocity measures is purely mechanical, and related to the structure of bees’ antennal muscles. When taking into account the known biological value of these odorants for bees, interesting general tendencies emerged. While the slow-backward movements were mostly expressed in response to alarm/defense pheromones (see red dots in Fig. 3C), especially to 2-heptanone, fast-forward movements were rather elicited by food-related odors (octanoic acid, the royal jelly odor, blue, and octanal, grey), pheromone components linked to the signaling of valuable resources (geraniol, an aggregation pheromone component, green), as well as social signals like brood and queen pheromones (light and dark violet, Fig. 3C). There were some exceptions to these rules, like the recorded backward antennal response to citral (an aggregation pheromone component) or β-ocimene (a volatile brood pheromone compound). Similarly, some odorants with a strong inferred biological value, like the fecal compound 3-methyl indole (scatol), did not induce strong antennal responses. This being said, fast forward movements to food-related odors appear consistent with previous studies showing that sucrose, or odorants previously associated with sucrose, induce forward antenna movements^50,52,56–58^. Such antennal responses are part of food-associated behavioral routines, together with extension of the proboscis. On the other hand, slow/backward antenna movements to alarm/defense compounds seems coherent with a defensive context, where responding to appetitive stimuli is of secondary importance, and protecting important sensory organs like the antennae may be more appropriate. In addition, the strong backward response to 2-heptanone somehow fits with its use by bees as a deterrent to mark depleted flowers^59,60^.

Clearly odorant quantity had an effect on antennal responses, because antennal position (but not velocity) varied as a function of odorant’s vapor pressure. When testing three odorants at eight different concentrations we found that both position and velocity varied with odorant concentration. Antennal responses generally started at 10^–3^ concentrations, which corresponds to concentrations at which clear odor-induced neural activity is observed in the antennal lobe in optical imaging experiments^61–63^. Interestingly, antennal responses did not simply increase monotonously with concentration. Remarkably, bees’ antennal responses to geraniol were stronger at medium than at high concentration. This possibly relates to the known dose-dependent effects of pheromones on behaviour^64–67^ and the fact that in natural situations, pheromones are used within a definite concentration range. It is thus possible that given concentrations best evoke odorants’ pheromonal value for bees and therefore trigger stronger antennal responses than higher concentrations. In any case, odorant concentration affected the amplitude of the response, but not its direction. We did not observe any opposite responses (forward/backward or slower/faster) for the same odorant at different concentrations.

Opposite influences of pheromones with differing biological values on antenna movements as observed in our experiments are remarkable in the context of current debates on pheromones’ behavioral side-effects. It has been increasingly suggested that pheromones, in insects but also in mammals, can act as modulators of a variety of behavioral responses which are not the primary – known—targets of their action^68,69^. In honey bees, some alarm pheromone components decrease bees’ responsiveness to an appetitive reward like sucrose^69^ and negatively impact appetitive learning performances^67,70–72^. Conversely, an aggregation pheromone component, typically associated with appetitive behavior, has been shown to decrease responsiveness to a noxious stimulus like an electric shock^73^ and to improve appetitive learning^72^. The model extracted from these findings posits that pheromones—or odorants with a strongly innately attached value—modulate the bees’ internal state relative to two main modules, an appetitive module and an aversive module (‘defensive and appetitive scores’^73^). It classifies pheromones in clear categories along a common hedonic dimension, with alarm pheromones bearing a negative (‘defensive’) value and aggregation pheromones (or floral odorants) bearing a positive (‘appetitive’) value. Accordingly, alarm pheromones reduce the appetitive score and aggregation pheromones reduce the defensive score. The contrasted antennal responses we observed may represent behavioral clues for the existence of such opposite odorant values. We attempted to capture such a hedonic dimension in our odorants by measuring their attractiveness for bees in an olfactory orientation setup (Fig. 4). However, correlation coefficients between attractiveness indices and antenna movement variables were not significant (p = 0.12 for both angle and velocity), even if the figures suggest a possible trend, with attractive odorants corresponding more to fast and forward antenna movements. Possibly, including more odorants in future studies could provide more statistical power for demonstrating a link between both variables. Note however, that the real-life situation is more complex than the simple hedonic model presented above, since not all pheromonal components of a given type have the same effect on behavioral responses. For instance, 2-heptanone, but not isopentyl acetate affects responsiveness to sucrose^69^, whereas isopentyl acetate, but not 2-heptanone, affects responsiveness to an electric shock^73^. Likewise, in our data, the brood pheromone β-ocimene brought the antennae to the back, while the other brood pheromones (ethyl oleate and methyl linoleate) brought them to the front (Fig. 3A). Thus, bees’ evaluation of odorants may be best described on more than one simple dimension, as each conveys a different message, usually presented in a particular context.

We evaluated the reproducibility of antennal responses to odorants by measuring them on bees from different hives on different years. We found a clear correlation between the two years’ datasets, both in terms of antenna position and velocity. This result may indicate that some of these responses (in particular to pheromones) are innate. Our observations suggest however a strong importance of bees’ experience. While some odorants induced very similar responses on both years, others induced remarkably different behaviors. Octanoic acid, for instance, produced strong forward and accelerated movements on the first year, but only weak responses on the second year. This points to an effect of experience, which we demonstrated in a previous study where odorants associated with food suddenly induced fast forward antennal movements^52^. In fact, finding the same pattern of odor-specific responses on the two years is not a proof per se that antennal responses are innate, because the observed patterns could simply be the result of our odorants being associated with similar contexts and consequences during the lives of these two groups of bees. To understand the ontogeny of these odor-induced responses, we recorded antenna movements in newly emerged bees. We found rather limited antennal responses at this age, especially regarding angular position changes. This suggests that odor-specific antenna movements are acquired by the bees in the course of their adult life. This could be due to an incomplete maturation of their antennal motor abilities when emerging, but could also relate to their behavioral development in the context of bees’ age polyethism. Interestingly, newly emerged bees did not show the specific slow-backward movements to alarm pheromones found in older bees. This is consistent with the fact that bees’ aggressiveness and response to alarm pheromones increases with age^74^, paralleling the ontogeny of defensive behavior^75,76^. This behavioral development is accompanied by changes in biogenic amine and hormone titers^77–79^. For instance, the levels of juvenile hormone and octopamine increase with age^80–82^. A role of biogenic amines in particular is supported by the observation that octopamine and serotonine have opposite effects on antennal movements, increasing and decreasing them respectively^58^. Thus, part of the age effect we found could be related to differences in levels of these biogenic amines. Our study thus gives some insights into the effect of age on odor-evoked antennal movements, but another aspect that would be extremely interesting to study is the effect of the bees’ tasks. Since younger individuals perform tasks within the colony while older individuals engage in tasks outside of the hive, we may consider that our experiments described the two ends of the task spectrum of a normal colony. Future experiments should however disentangle the respective effects of age and task on antennal responses to task-related odors and pheromones.

Different odorants induce different antenna movements according to their biological value for bees. This, together with the observation that antennal movements are modified by associative conditioning^52^, suggests that antennal movements are under central top-down modulation. The movements of the antennae are controlled by different muscle groups moving the antenna scape (4 muscles) and the flagellum (2 muscles). Motor neurons controlling this muscular system originate in the AMMC (antenna mechanosensory and motor center)^83–86^. Response to tactile stimuli is thought to use a short route as mechanosensory neurons project directly to the AMMC^83^. Antennal reaction to olfactory stimuli, by contrast, should take a longer route. Odorants are detected by olfactory sensory neurons in the antenna, which relay odor information to a primary olfactory centre, the antennal lobe (AL), composed of glomeruli, which each receives input from OSNs (Olfactory Sensory Neurons) expressing the same olfactory receptor type. The AL processes olfactory information and second-order (projection) neurons (PN) transmits it to higher-order brain centers, the mushroom bodies (MB) and the lateral horn (LH). Honey bee pheromone compounds (alarm, aggregation, brood, queen, etc.) all trigger combinatorial activity from many glomeruli in the worker antennal lobe^87,88^. This suggests that their biological value is extracted within higher-order centers^89^. Indeed, projections from the AL to the honey bee LH were shown to contain combinatorial information allowing to differentiate the different pheromone types^90^. The LH is considered as a premotor center mediating fast and innate reactions to biologically relevant stimuli, and may be responsible for innate antennal movements to odorants. Direct connections between the LH and the AMMC are not described yet in honey bees, but they are known in fruit flies^91,92^. Changes in odor-induced antennal responses through experience (like after associative conditioning) would involve the MB, the learning and memory center of the insect brain. In the MB, the Kenyon cells (KC) are highly odor-specific and are activated by the combinatorial input from many different PNs^93^. Information from KCs is read out by MB-output neurons which project to different parts of the protocerebrum, including the LH. MB-output neurons are plastic and their odor-induced responses are modified by experience^94–97^. Some of them, like the PE1 neuron, project to the LH^95,98^ and may be responsible for an experience-driven modulation of odor-induced antennal movements through this structure. To our knowledge, no direct connections of MB output neurons to the AMMC have been described^98^, but indirect pathways other than through the LH are possible.

To conclude, honey bees display a range of different antennal responses to odorants, which vary as a function of odorants’ biological value. These responses are reproducible, suggesting that they are in part innate, but they are also shaped by the bees’ experience and develop in the course of their lives. A necessity of our approach, but a clear limitation nonetheless, is that bees’ responses were recorded in an experimental context quite different from natural situations. In other insects, social context in particular has been shown to modulate behavioral responses to olfactory stimuli^99,100^. Bees’ antennal response to alarm pheromones, for instance, may be quite different when they are guarding at the hive entrance. A next step should thus be to analyze antennal movements in more natural, hive, situations.

## STAR★methods

The material used in this article is listed in Table 2.

**Table 2:** Key resources table.

Reagent or resource	Source	Identifier
**Experimental models: organisms**		
Honeybee *Apis mellifera*	CNRS Gif-sur-Yvette	N/A
**Software and algorithms**		
Brain Vision Systems	http://www.bvs-tech.com/website/eng/index.php	BIPcam
Statistica®	StatSoft, Inc. 2004	Statistica 7.0
**Other**		
Posca pen	https://www.mba-shop.com	Posca PC-5 M, Mitsubishi Pencil Co
White light source	Leica, Jena, Germany	CLS 150XE
Low-temperature melting wax	www.kerrdental.com/kerr-laboratory/utility-wax-waxes	09,731

### Contact for reagent and resource sharing

Further information and requests for resources, data and reagents should be directed to and will be fulfilled by the Lead Contact, Jean-Christophe Sandoz (sandoz@egce.cnrs-gif.fr) and Hanna Cholé (hanna.chole@gmail.com).

### Experimental model and subject details

The experiments were carried out on adult honey bee workers (*Apis mellifera*) captured at the hive entrance on the CNRS campus in Gif-sur-Yvette (France) in April–May 2015 and 2016. The newly emerged bees used in the last experiment (Fig. 6) were caught on the morning of the experiment when emerging from a brood comb placed the day before in an incubator (at 35 °C). Bees were chilled on ice until they stopped moving in order to harness them individually in plastic holders, leaving their antennae and mouthparts free. They were then fed with 5 µl sucrose solution (50% w/w), 4 h before the beginning of the experiments. After feeding, a drop of color was applied on bees’ antennae tips, using water-based paint (Posca PC-5 M, Mitsubishi Pencil Co.). Bees were marked on the upper surface of the last two flagellomeres, to allow the motion capture system to record their coordinates (see below), without impairing olfactory perception see^52^. Once mounted, fed and marked, individuals were placed in a moist, dark polystyrene box, until the start of the experiments.

## Method details

### Antenna monitoring apparatus

The recording apparatus was composed of a camera positioned above the bee holder (Fig. 1A). The camera included an integrated processing card allowing adaptive detection (using a motion prediction algorithm) of the two color dots, up to a rate of 120 Hz (BIPcam, Brain Vision Systems). The camera managed to follow and record the coordinates of the color dots on the antenna tips, in real time at a rate of 90 Hz. In order to optimize the detection of the color dots, the apparatus was placed in a room with low light conditions (controlled and kept constant). A cold light illumination ring was placed around the lens of the camera, diffusing homogeneous white light on the bee’s head (Leica CLS 150XE, Leica, Jena, Germany). The intensity of the light source was tuned precisely and kept constant for the duration of the experiments.

The olfactory stimulation apparatus provided a constant air flow of 52.5 mL/s. This flow, composed of a principal air flow of 50 mL/s and a secondary flow of 2.5 mL/s, was directed to the bee by a glass tube (0.5 cm diameter), at a distance of 2 cm. The secondary air flow could be directed to one of two sub-circuits (one containing an odorant source, and another without any odorant) before being reinjected into the main airflow. Most of the time, air flowed through the odourless sub-circuit. Olfactory stimulation was applied manually inducing a switch of the secondary flow to the odorant sub-circuit for 5 s. The odorant sub-circuit included a Pasteur pipette containing the odor source (see below). The other sub-circuit included an identical Pasteur pipette without odorant. An air extractor, placed behind the bee prevented odorant accumulation.

### Antenna movement analysis

Before the recording period, each bee was left to acclimatize for 20 s to the apparatus. Each recording lasted 60 s. The monitoring apparatus recorded at each time point (90 times per second) the location of the two antenna tips of each bee on the camera sensor (pixel coordinates). First, all the recordings from all bees were recalculated in the same coordinate system (x,y), with the socket of the right antenna as the origin (coordinate 0,0) and the socket of the left antenna as the unit reference on the x-axis (coordinate 1,0). Each recording thus resulted in a series of (x,y) coordinates for each antenna at each time-step (1/90 s). This allowed a comparison between the antennal movements of different bees.

Previous studies^51,52^ showed that bees’ antennal movements are best described using circular coordinates (r, θ), as each antenna moves around its socket (Fig. 1B). Thus, each antenna’s movements were described in their own coordinate system, with the antenna socket (base) as the origin (0,0).

- Angular position (θ): it was defined as the angle between a line connecting the antenna tips to their base (r) and an anteroposterior line passing through the corresponding antenna base. This variable indicates if the antenna is positioned to the front (0°), to the side (90°) or backward (180°). Note that the measured angle is symmetrical for the left or the right antenna so that 90° is on the left for the left antenna and on the right for the right antenna.
- Distance to antenna base (r): it was defined as the distance between the antenna base and the antenna tip. This variable thus measures whether the antenna is in a stretched or retracted position.
- Angular velocity (Vθ): it was calculated as the angle θ traveled by each antenna during a frame (1/90 s). It is expressed in degrees per second.

As explained in the results, θ and Vθ proved to be the most pertinent for measuring changes induced by conditioning and are thus presented in the figures. The r data are presented in Supplemental Material (Suppl. Fig S1). The response to each odorant was calculated as the change between the antennal movements before and during the odorant stimulation. Thus, Δθ (resp. ΔVθ) was calculated as the average of θ (resp. ΔVθ) during the stimulus minus the average of θ (resp. ΔVθ) before the stimulus (Fig. 1C,D).

In most experiments, the two antennae of the bees were marked and recorded, and angle and velocity data were averaged between antennae before any analysis. In the concentration experiment (Fig. 6), only one antenna per bee (balanced between right and left) was tracked allowing faster data analysis. Since previous studies observed different behavioral and/or neurophysiological effects depending on the side of olfactory stimulation (through the right or left antenna^116–118^, we statistically assessed whether antenna movements differed between sides. We found no effect of antenna side, neither for Δθ (RM-ANOVA, F_1,41_ < 3.29, p > 0.077) nor for ΔVθ (RM-ANOVA, F_1,41_ < 0.31, p > 0.579).

### Odorants for antenna movement analysis

Up to fifteen odorants with different biological values for bees were tested in the experiments. They are detailed in Table 1. For most stimuli, 5 μL of the odor solution were placed on a filter paper strip inserted in a Pasteur pipette. The odorants were used pure except for 3-methyl indole, which was a powder diluted in water at a concentration of 0.48 mg L^−1^^119^. The queen pheromone QMP was presented as a commercial stick (BeeBoost ®, Pherotech, Delta, Canada), directly inserted into a Pasteur pipette. Except for this last stimulus, all odorants were obtained from Sigma-Aldrich (Deisenhofen, Germany). As control stimulus, a pipette containing a clean piece of filter paper was used. The order of odorant presentations was randomized between bees. In one experiment, three odorants (geraniol, octanoic acid and 2-heptanone) were tested at eight concentrations ranging from 10^–7^ to 10^0^. They were prepared in mineral oil and were presented in increasing concentration order to avoid adaptation (i.e. the 10^–7^ concentrations of the three odorants were presented to the bee before moving to the 10^–6^ concentrations, etc.). The control stimuli were a pipette containing a piece of filter paper soaked with 5 μL mineral oil and a pipette with a clean piece of filter paper. Both were presented before and after the odorant concentrations. The interval between odor presentations was ~ 60 s.

### Odorant attractiveness measure

To determine the attractiveness of each odorant for bees, we developed a high-throughput assay allowing to simultaneously monitor the movements of 16 bees, each confronted to a different stimulus (Fig. 4A). Each of the 16 lines of the setup was 45 cm long, made of 3 connected glass tubes (12 mm internal diameter), with two plastic (3D printed) boxes placed at each end, one containing the bee and the other containing a filter paper with 5 µL of pure odorant. The bee box contained a sliding door allowing to control when each bee was allowed to enter the glass tube. Three automatic, equally spaced infra-red portals (Trikinetics, Walham, MA, USA), allowed counting the passages of each bee at three locations along the tube. The apparatus was placed in the dark to ensure that bees’ movements were based on olfaction, and under an air extractor to prevent any accumulation and contamination of odorants. Sixteen bees were tested simultaneously, each with one of the 15 odorants or with an odorless box (control). Each bee was allowed to enter its tube by opening the door of its box. Her movements through the three portals were then monitored for 10 min. Bee boxes and the glassware were thoroughly washed between trials, while odorant boxes were kept separate in plastic bags and always contained the same odorant throughout the experiment.

In the end, an orientation index was calculated from the number of passages through the portals using the following formula: (M3-M1)/(M3 + M1), were M1 is the monitor close to the initial position of the bee, the farthest from the odorant box, and M3 is the monitor the closest to the odorant box. Each bee was used only once in the apparatus, with only one odor. The raw position index of the control group (odorless box) was subtracted from that of each odorant to obtain this odorant’s attractiveness index (AI). A positive AI (Fig. 4) thus indicated an attractive odor whereas a negative AI indicated a repellent odor.

### Statistical analysis

Before analysis, the normal distribution of antennal and attractiveness data were confirmed with Shapiro-Wilks tests. To compare antennal responses (changes in angular position Δθ and velocity ΔVθ) among odorants, repeated measure analyses of variance (RM-ANOVA) were used, with *stimulus* (odorants including air control) as within group factor. When significant, Dunnett post-hoct tests allowed to compare each odorant’s value to the control. In the odorant concentration experiment, a RM-ANOVA with *stimulus* and *concentration* as within-group factors was used. If significant, it was followed by individual RM-ANOVAs for each odorant, with only *concentration* as a within-group factor. When significant, individual concentrations were compared to a common control (average of the four air/mineral oil stimulations), using Dunnett tests. To make sure there were no difference between the groups marked in the right or left antenna, a RM-ANOVA with *antenna side* as categorical factor was used, on all the odors and concentrations, as well as on the controls separately. Concerning the odorant attraction assay, differences in attractiveness index were compared among stimuli using an ANOVA (note that different bees were tested with each odorant). Pearson correlation tests were used to evaluate the relationships between the attractiveness index, odorants’ vapor pressure or concentration and the antennal movement variables Δθ and ΔVθ. All statistical analyses were performed with Statistica® 7.0 software (StatSoft, Inc. 2004).

## Supplementary Information

Supplementary Information.

## Data Availability

The data are available on request from the authors.
