# Supplementary material for: Antenna movements as a function of odorants’ biological value in honeybees (Apis mellifera L.)
Source: Sci Rep. 2022 Jul 8;12:11674. doi: 10.1038/s41598-022-14354-z (PMC9270438; doi:10.1038/s41598-022-14354-z)
Supplement: Supplementary file 1 — Supplementary Information. [file 41598_2022_14354_MOESM1_ESM.docx]

**Supplementary Figure legends:**

**
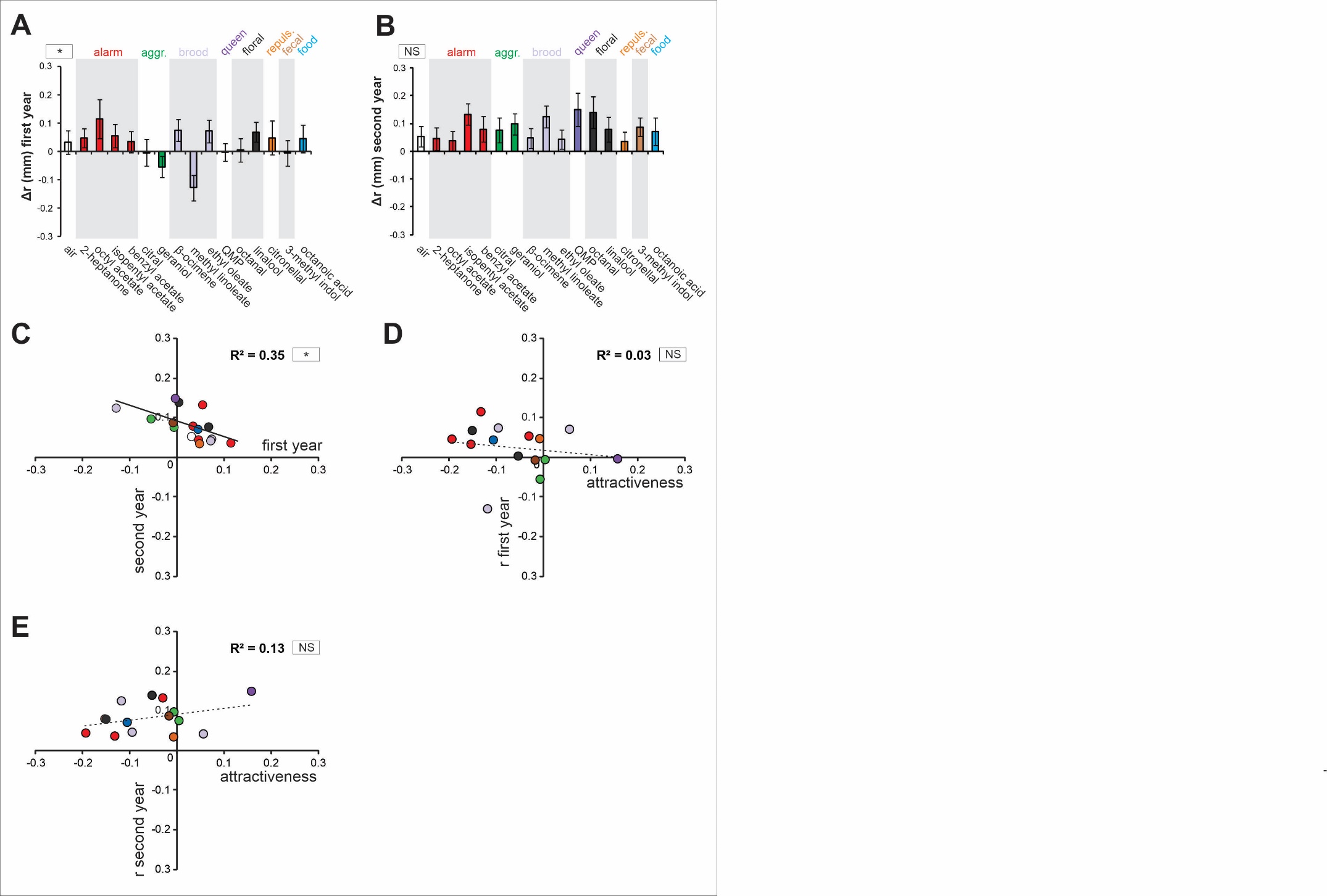
**

**Supplementary Figure 1: Influence of odorant identity on the distance between antenna tip and base (r).** Among the antennal variables calculated in Fig.1B, the distance to antenna base (r = radius) showed low variation in response to the odorants (first year: RM-ANOVA; *stimulus* effect; F_15, 345_ = 1,91; p = 0.021; Dunnett test NS; second year: RM-ANOVA; stimulus effect: F_15, 360_ = 1,12; NS). We thus focused the analyses on antenna angular position and velocity in the main text. **A-B.** Histograms showing the change of antennal movements in response to odor presentation (during–before odor) in terms of distance from the antenna tip to the base (r) on the (A) first year and (B) the second year. Color code: air control (white), alarm pheromones (red), aggregation pheromones (green), brood pheromones (light purple), queen pheromone (dark purple), floral odors (grey), repulsive odor (orange), fecal odor (brown), and the royal jelly component (blue). Asterisks in the square next to the graph indicate a significant heterogeneity in antennal movements between odorants (RM-ANOVA, *: p < 0.05). NS: non-significant. **C.** Regression comparing the results of the two experimental years in terms of distance from the antenna tip to the base (r). Stars in the square next to the graph indicate a significant Pearson correlation (*: p < 0.05). We have no explanation for this (possibly spurious) negative correlation between r values on the two years.


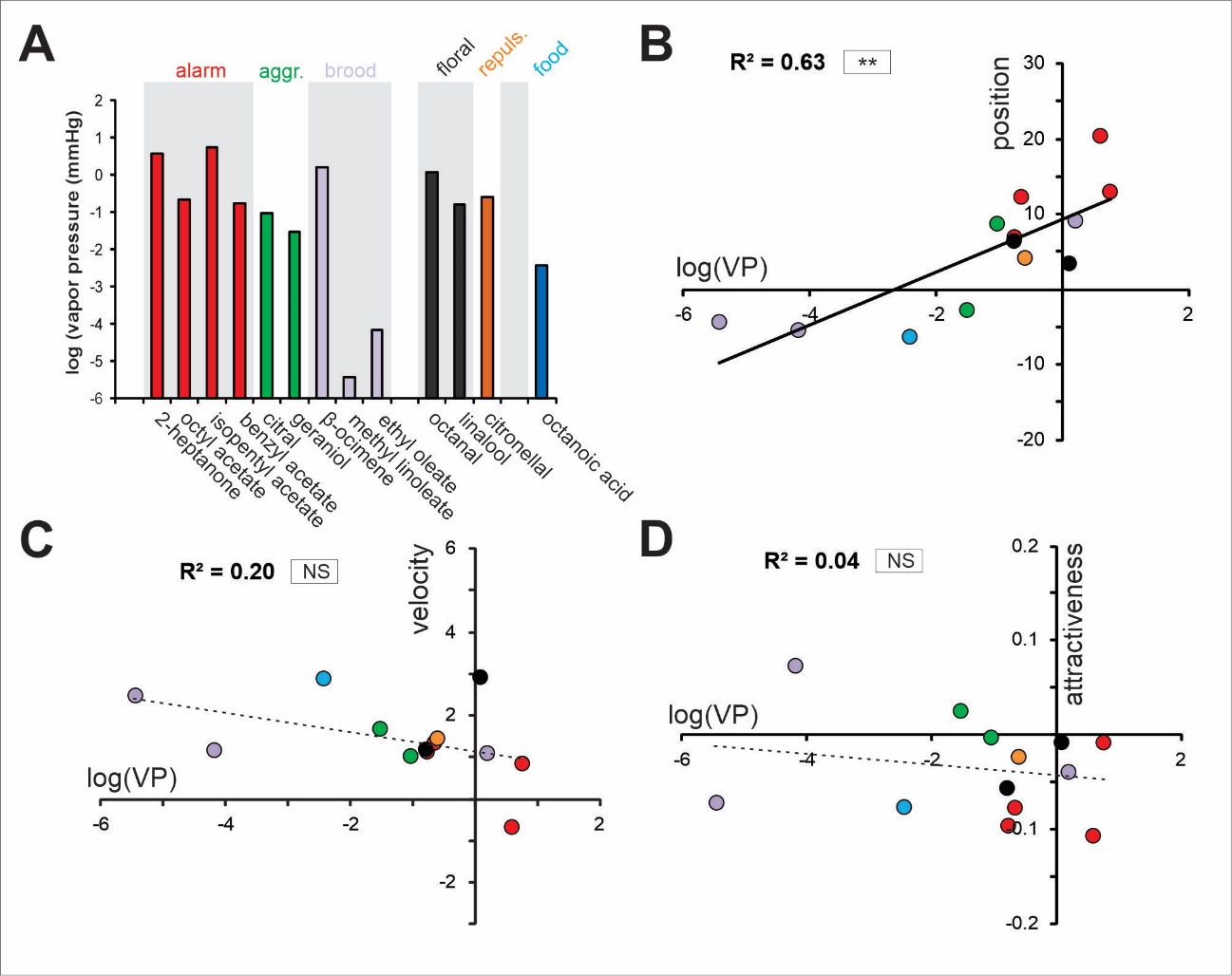


**Supplementary Figure 2: Effect of odorant vapor pressure on odor-induced antennal movements and odorant attractiveness. A.** Histograms showing the vapor pressures at 25°C for each odorant (mm Hg in log scale). QMP and 3-methyl-indol were excluded from these analyses as they are respectively a mix of several odorants and a powder dissolved in water. **B-D.** Regressions presenting (B) changes in antennal angular position (Δθ), (C) changes in antennal velocity and (D) odorant attractiveness as a function of odorants’ vapor pressure. Asterisks in the square next to each graph indicate a significant Pearson correlation (**: p < 0.01). NS: non-significant.
